# Nanotechnology in the perioperative treatment of head and neck cancer: application and outlook

**DOI:** 10.3389/fbioe.2025.1566522

**Published:** 2025-03-27

**Authors:** Jun-Jie Zhou, Yan-Chuan Feng, Min-Long Zhao, Qi Guo, Xi-Bo Zhao

**Affiliations:** The Stomatological Hospital, Anyang Sixth People’s Hospital, Anyang, China

**Keywords:** head and neck cancer, perioperative period, nanotechnology, diagnosis, treatment

## Abstract

Head and neck cancer (HNC) critically affects patient survival and quality of life, highlighting the need for optimized perioperative interventions. While conventional therapies face limitations in specificity and toxicity, nanotechnology has emerged as a revolutionary approach. Preoperative application of tumor-targeting nanoprobes enables molecular-level lesion identification *via* biomarker-specific conjugation and spatially resolved fluorescence quantification. Intraoperatively, fluorescent nanomaterials enhance surgical precision through selective tumor accumulation, delineating malignant margins in real time. Postoperatively, engineered nanocarriers improve therapeutic outcomes by delivering drugs with spatial control, minimizing off-target effects, and enabling multimodal synergies. These nanotechnology-driven strategies collectively address critical challenges in HNC management, including diagnostic sensitivity, intraoperative visualization, and postoperative recurrence. Their inherent advantages—precision targeting, reduced systemic toxicity, and multifunctional integration—establish them as cornerstone tools in modern oncology. Future advancements in nanomaterial design and biocompatibility are poised to further refine therapeutic efficacy, survival rates, and patient-centered outcomes.

## 1 Introduction

Head and neck cancer (HNC) represents a major global health burden, with incidence rates steadily increasing worldwide (Stransky et al., 2011; Chow and Longo, 2020; Mody et al., 2021). According to the data collected by the World Health Organization, the annual number of newly diagnosed HNC cases across the globe exceeds 600,000, with approximately 300,000 deaths each year (Bray et al., 2024). The occurrence of HNC is associated with a multitude of factors (Leemans et al., 2018; Cramer et al., 2019; Galizia et al., 2022). These include behaviors such as smoking and excessive drinking, as well as the intake of betel nuts (Galizia et al., 2022). Additionally, infection with the human papillomavirus (HPV) is also identified as a contributing factor (Sabatini and Chiocca, 2019). In China, the incidence of HNC is notably high (Barsouk et al., 2023). This is particularly evident in certain regions, such as Hunan and Guangdong provinces, where the extensive consumption of betel nuts has led to a continually increasing incidence rate (Hu et al., 2017a). HNC has a deep impact on patients. It not only impairs the normal physiological functions of the oral cavity, including mastication, swallowing, and the ability to express speech, but also induces significant changes in patients’ physical appearance (Chen et al., 2014). These consequences collectively subject patients to substantial physical and psychological distress (Borggreven et al., 2005; Chen et al., 2014). Hence, the quest for strategies to improve the therapeutic outcomes of HNC and to enhance the survival prospects and quality of life of affected individuals has emerged as a notable area of focus within the medical research community (Miller and Shuman, 2016; Machiels et al., 2020).

The traditional treatment methods for HNC mainly include surgery, radiotherapy, and chemotherapy, etc (Miller and Shuman, 2016; Lechner et al., 2022; Elaheh Dalir et al., 2023). Surgery is the primary approach for treating HNC, aiming to achieve the therapeutic goal by removing the tumor tissues (Ferris et al., 2016; Burtness et al., 2019; Gillison et al., 2019). However, this approach is constrained by incomplete resection, high recurrence rates, and it can have a significant impact on patients’ oral functions and appearances (Ferris et al., 2016; Burtness et al., 2019; Gillison et al., 2019). Radiotherapy and chemotherapy, as adjuvant therapies for surgery, can improve the treatment outcomes (Gyawali et al., 2016). However, they also come with some side effects (Gyawali et al., 2016; Burtness et al., 2019; Gillison et al., 2019; Lechner et al., 2022). Radiotherapy may lead to oral mucosal damage, dry mouth, and hypogeusia, while chemotherapy can cause nausea, vomiting, hair loss, and bone marrow suppression (Mallick et al., 2015; Zhang et al., 2024). In addition, the treatment effects of traditional methods on some patients with advanced HNC are not satisfactory, and the survival rates and quality of life of these patients remain relatively low (de Roest et al., 2022; Goel et al., 2022; Lee et al., 2024). Given these limitations, there is an urgent need for innovative strategies that can enhance therapeutic precision while minimizing adverse effects. This has driven significant interest in nanomaterials and other advanced approaches, such as bioengineered hydrogels (Adine et al., 2024; Nie et al., 2025), which demonstrate promising potential in improving drug delivery, promoting tissue regeneration, and modulating the tumor microenvironment in HNC (Wu Y. et al., 2023; Zang et al., 2024).

**FIGURE 1:**
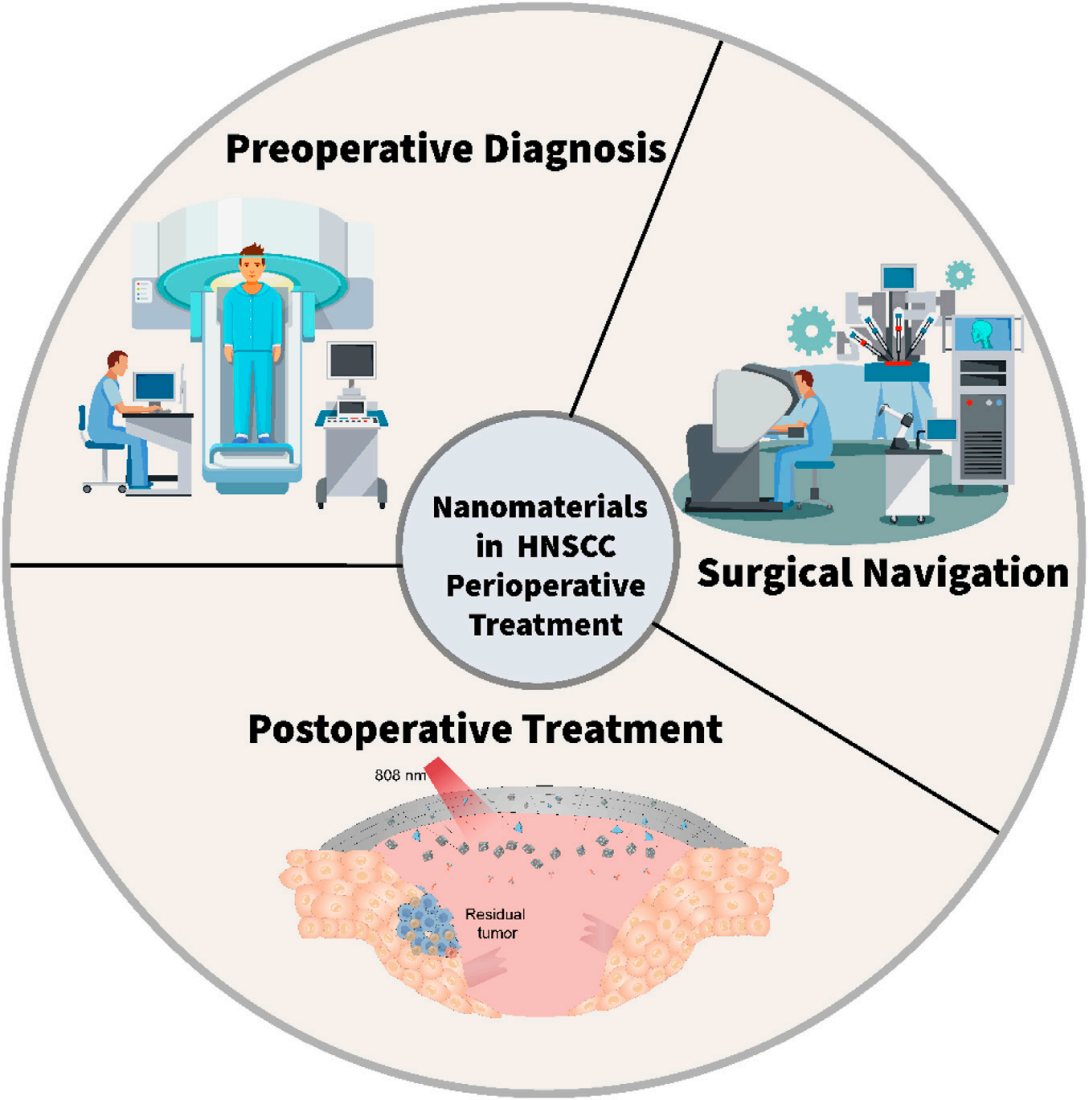
The scheme illustrates the application of nanomaterials for the perioperative management of HNC.

Nanotechnology is an emerging technology that involves the control of substances at the nanoscale (Chaturvedi et al., 2019; Chehelgerdi et al., 2023; Malik et al., 2023). The application of nanomaterials in the medical field has brought new hopes for the treatment of HNC (Xu et al., 2022; Wu Q. et al., 2023; Tang et al., 2024; Wang et al., 2025). The unique advantages of nanomaterials include: (1) The size of nanomaterials generally ranges from 1–100 nm, which is similar to the size of biomolecules and cells (Shang et al., 2014; Navya et al., 2019; Liu R. et al., 2023). Thus, they can more easily penetrate cells and tissues, enabling precise treatment of tumor cells (Sykes et al., 2014; Shi et al., 2020; Lammers, 2024; Wang B. et al., 2024). (2) Nanomaterials have a relatively large specific surface area, which can increase drug loading capacity and the interaction with biomolecules, enhancing the efficacy of drugs (Xu et al., 2022; Tang et al., 2024; Wang et al., 2025). (3) Nanomaterials can be surface-modified with targeting molecules to achieve specific recognition and targeted treatment of tumor cells, thereby reducing damage to normal tissues (Hong et al., 2016; Zhu and Li, 2023). (4) Nanomaterials can be chemically modified and physically modified to endow them with various functions, such as fluorescence imaging, magnetic resonance imaging, photothermal therapy, *etc.*, enabling the integration of tumor diagnosis and treatment (Smith and Gambhir, 2017; Liu et al., 2019; An et al., 2021). During the preoperative phase, nanomaterials enable precise diagnosis and targeted therapy, enhancing treatment efficacy and minimizing adverse effects. During surgery, nanoprobes facilitate real-time imaging and monitoring, assisting surgeons in achieving accurate tumor resection. Postoperatively, nanocarriers can deliver sustained-release drugs locally to prevent recurrence. This review systematically examines the advancements in nanomaterials for the perioperative management of HNC, focusing on three key aspects: preoperative diagnosis, intraoperative imaging, and postoperative recurrence management (Figure 1). By exploring these applications, we aim to provide new perspectives and strategies for improving HNC treatment outcomes and patient prognosis.

## 2 The application of nanomaterials in the preoperative diagnosis and treatment of HNC

### 2.1 Nanoprobes are applied in the early diagnosis

#### 2.1.1 Quantum dot probes

**FIGURE 2:**
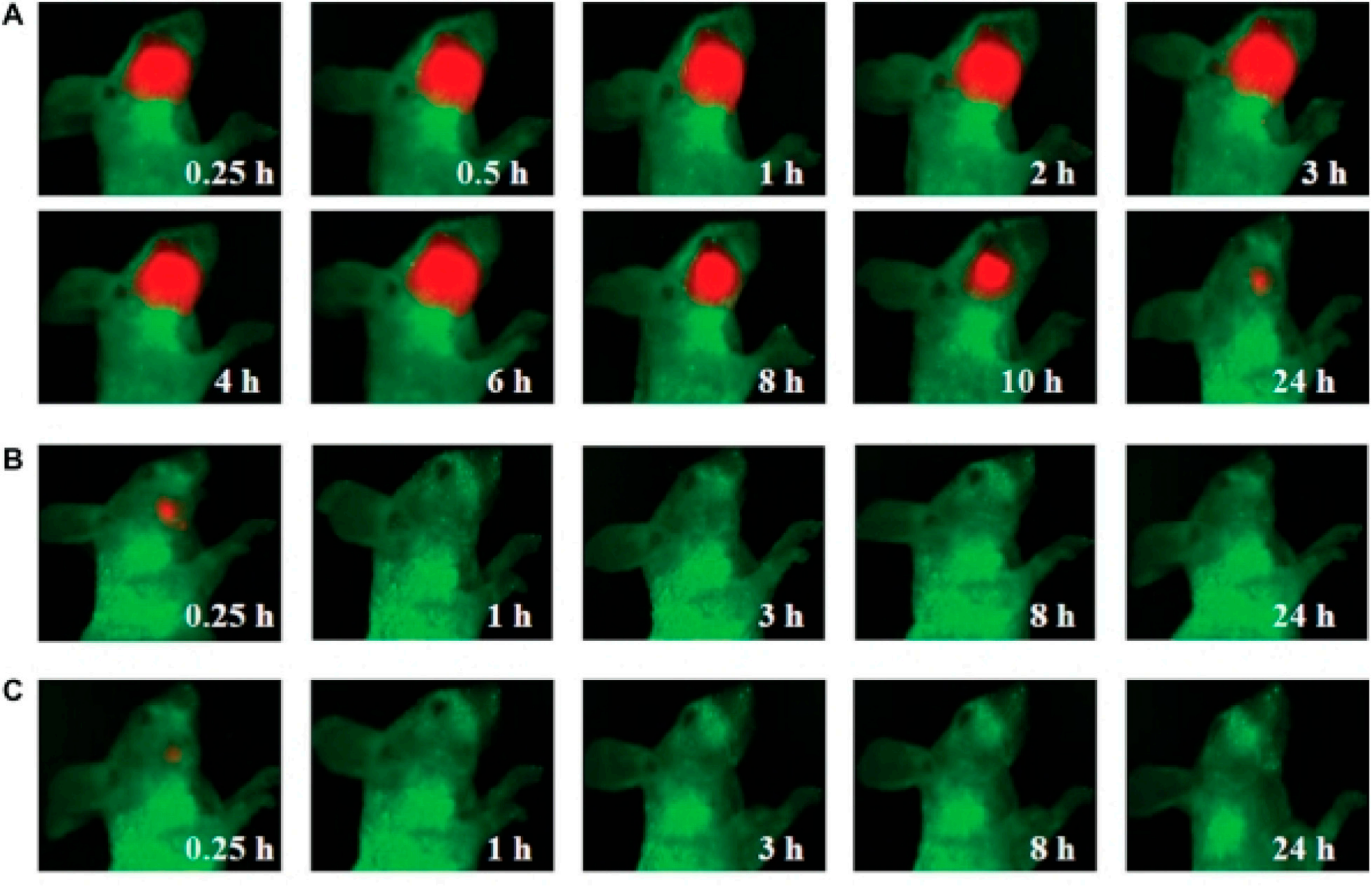
Visible imaging of HNC animal model by QDs probe **(A)** Visible images of HNC animal model after intravenous injection with QD800-EGFR Ab probe containing 100 pmol equivalent of QD800 **(B)** Visible images of HNC animal model after intravenous injection with 100 pmol QD800 **(C)** Visible images of HNC animal model after intravenous injection with 250 µL of EGFR monoclonal antibody (1 mg/mL). Reprinted with permission from Yang (2011). Copyright 2011 Dovepress.

Quantum dots (QDs), as a kind of semiconductor nanocrystals, exhibit distinctive optical traits, high fluorescence intensity, excellent stability, and adjustable emission wavelengths (Moon et al., 2019; Gidwani et al., 2021; Shetty et al., 2024). Notably, the fluorescence emission wavelength of QDs is size-dependent, meaning that through exact control of their size, the emission of fluorescence across various wavelengths can be achieved (Fang et al., 2017). These remarkable properties make QDs to be the highly suitable biomarkers for the early detection of HNC (Shetty et al., 2024). The function of QD probes is to attach to the specific markers on the surface of HNC cells, thus contributing to the early diagnosis of this malignancy. For instance, Ya-Dong Li’s group has designed a QD-based probe that has the ability to bind to the epidermal growth factor receptor (EGFR) on the cell surface of HNC (Figure 2) (Yang, 2011). By monitoring the fluorescence signal emitted by the QDs, an early diagnosis of HNC can be effectively achieved. This application offers a novel approach to the early diagnosis of HNC, potentially enhancing the overall diagnostic accuracy and patient outcomes. The application of QD probes holds great promise in the field of HNC diagnosis, opening up new ways for more sensitive and specific detection methods (Xu et al., 2012; Yakavets et al., 2020).

#### 2.1.2 Nanomaterial-based biosensors

**FIGURE 3:**
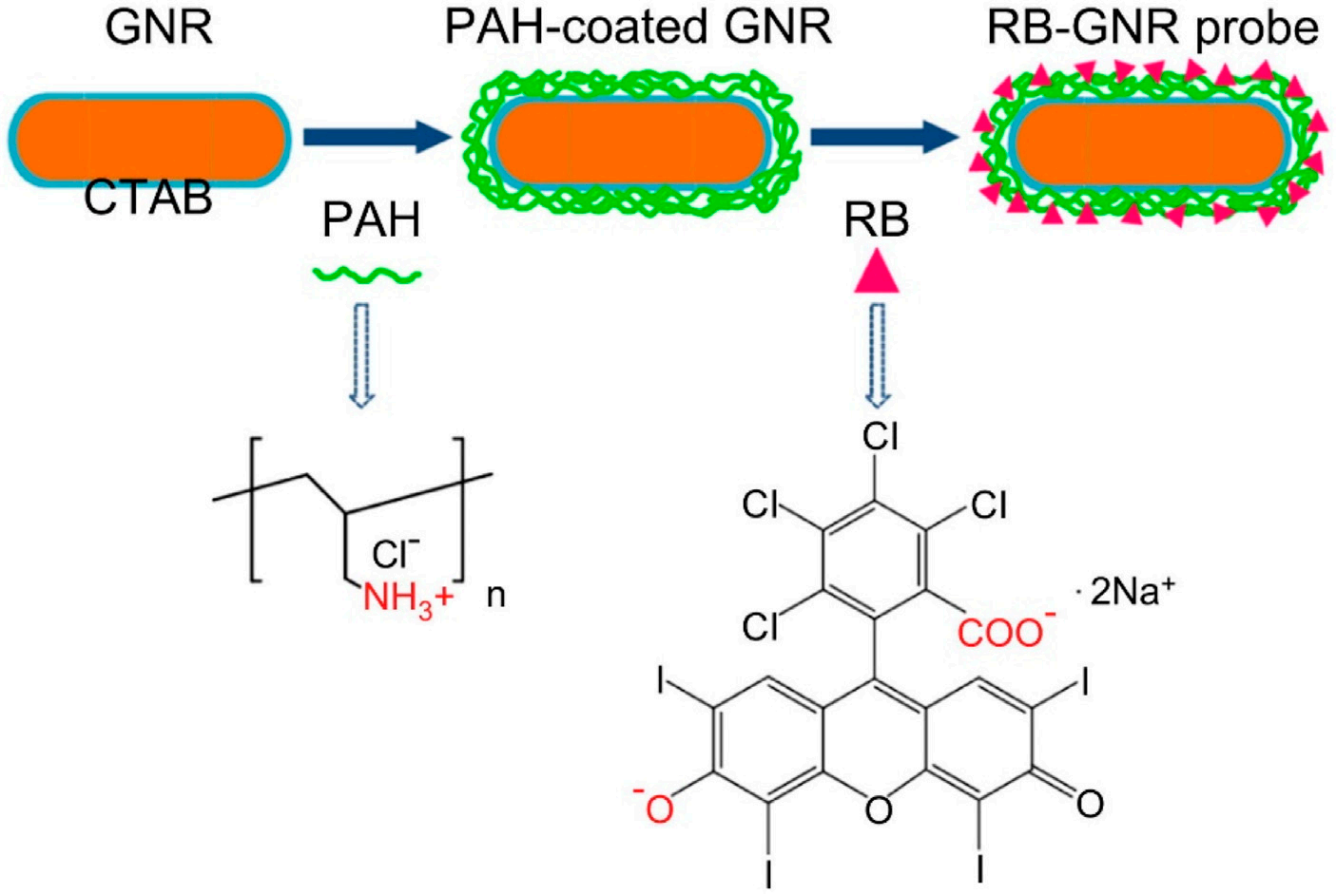
Schematic illustration of the synthesis of RB-GNRs. Reprinted with permission from Wang et al. (2013). Copyright 2023 Elsevier.

There are many kinds of nanomaterials used for constructing biosensors, such as metal nanoparticles, carbon nanomaterials, QDs, etc (Wong et al., 2020; Eivazzadeh-Keihan et al., 2022; Barhoum et al., 2023). These nanomaterials possess different properties and can be selected and constructed based on specific needs (Wong et al., 2020). For example, metal nanoparticles have excellent electrical conductivity and catalytic performance and can be used to construct electrochemical sensors (Azimzadeh et al., 2016). Carbon nanomaterials have good biocompatibility and electrical conductivity and can be used to build biosensors (Maiti et al., 2019; Mukherjee et al., 2024). Among them, biosensors achieve early diagnosis of HNC by interacting with HNC-related biomolecules to produce signal changes (Xue et al., 2018). For instance, a biosensor based on gold nanoparticles can bind to Rose Bengal (RB), which has been shown to have specificity for oral cancer through inhibition of DNA polymerase of HNC cells (RB-GNR) (Figure 3) (Wang et al., 2013). The RB-GNR platform has been tested on HNC cells, showing its dual functionality as both a sensor and an imaging probe. The sensing assay exhibits enhanced sensitivity and accuracy, while the imaging approach enables rapid, multi-channel detection. Both modes are highly efficient and convenient, highlighting the RB-GNR platform’s significant potential for cost-effective diagnostics in HNC.

### 2.2 The application of nanomaterials in defining the tumor boundary

#### 2.2.1 Targeted nanoparticle imaging

**FIGURE 4:**
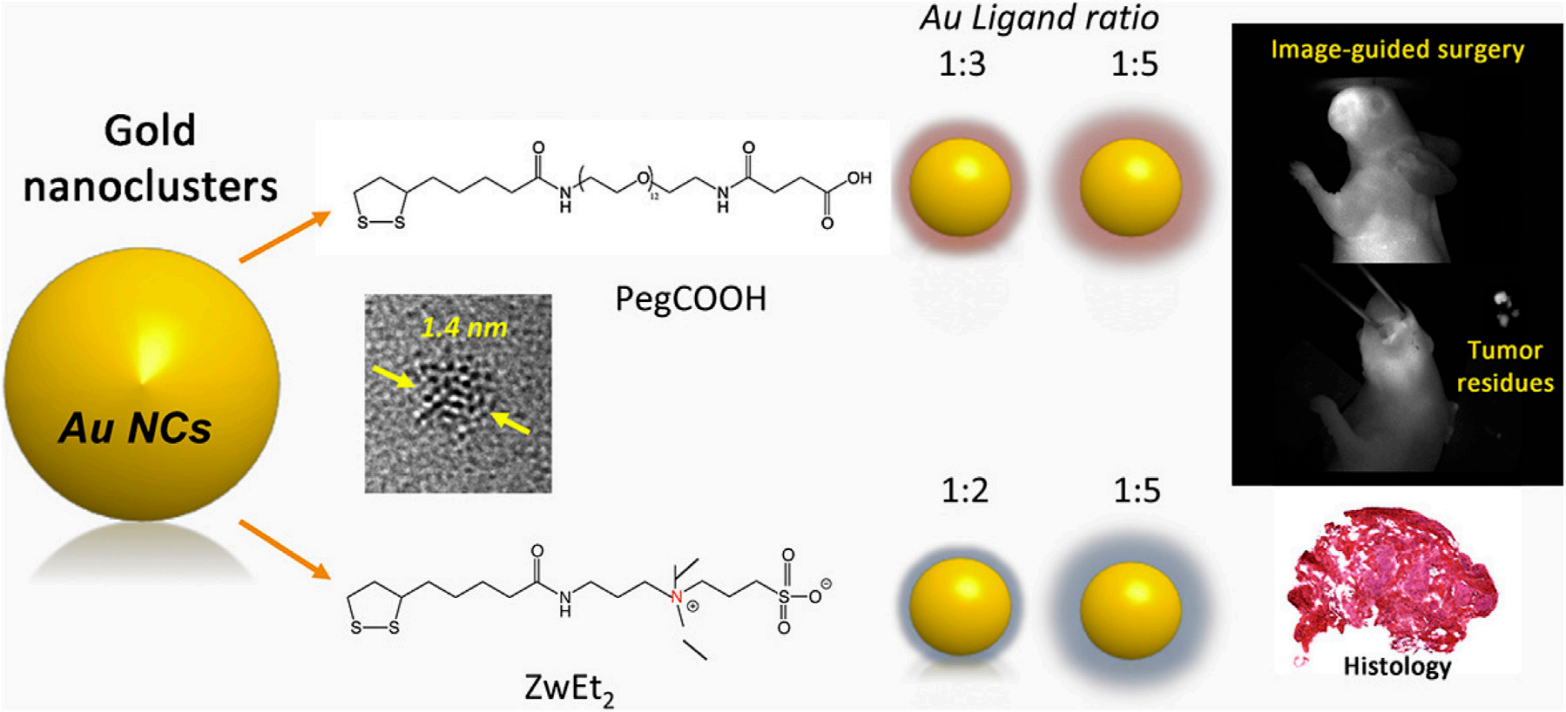
Schematic illustration of the designed AuNCs for image-guided surgery. Reprinted with permission from Colombé et al. (2019). Copyright 2019 Elsevier.

Targeted nanoparticle imaging requires the selection of targeting molecules that are specific to the markers of HNC cells (Wiechec et al., 2017; Zhao et al., 2019; Chen et al., 2023). These markers include receptors, antigens, enzymes, *etc.* For example, for EGFR on the surface of HNC cells, anti-EGFR antibodies can be selected as the targeting molecules (Sacco and Cohen, 2015; Wiechec et al., 2017; Bhatia and Burtness, 2023). The enrichment mechanism of targeted nanoparticles in HNC tissues is mainly through the specific binding of the targeting molecules to the markers on the surface of cancer cells (Rahman et al., 2012; Colecchia et al., 2017; Huang et al., 2022). HNC presents significant surgical challenges, as it requires the preservation of healthy structures while ensuring complete tumor resection (Chow and Longo, 2020; Mody et al., 2021). Image-guided surgery has emerged as a promising approach to enhance tumor removal precision (Neilio and Ginat, 2024). Near-infrared (NIR) imaging-guided surgery, in particular, has been successfully applied in both small animal studies and clinical trials (Vahrmeijer et al., 2013; Li et al., 2021). Among the various advancements, gold nanoclusters (AuNCs) have shown great potential as renal-clearable nanoplatforms, offering promising applications in tumor diagnosis and therapy. For example, Cindy Colombé has synthesized AuNCs with different ligands and tested them *in vitro* and *in vivo* (Figure 4) (Colombé et al., 2019). They demonstrated that AuNCs exhibit multifunctional potential in various applications, including optical imaging, enhanced radiotherapy, and photothermal therapy. The combination of image-guided surgery with these therapeutic modalities could significantly improve treatment outcomes. Furthermore, the development of AuNCs with short-wave infrared (SWIR) emission and high quantum yield holds promise for further enhancing the contrast between tumor tissue and healthy tissue, potentially revolutionizing precision medicine approaches. These imaging techniques can provide important reference bases for surgical planning and improve the accuracy of surgeries.

#### 2.2.2 Multimodal nanoprobes

**FIGURE 5:**
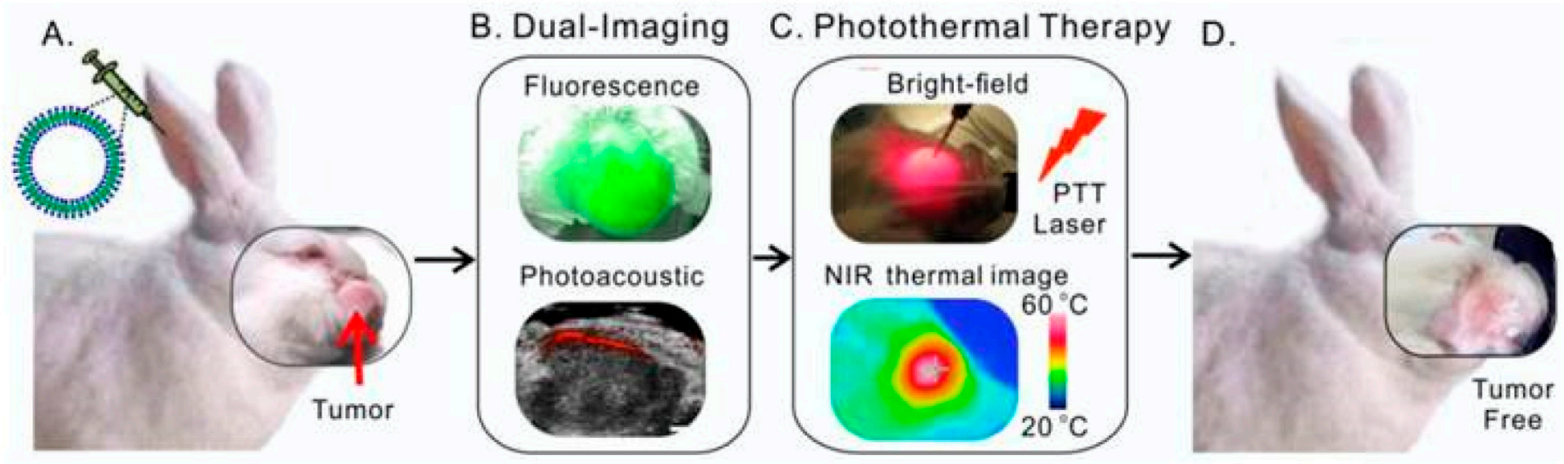
Schematic illustration of multifunctional Porphysomes for rabbit HNC. **(A)** Intravenous administration of porphysomes via the rabbit ear vein. **(B)** Both fluorescence and photoacoustic imaging were enabled by porphysomes accumulated at 24 h post-injection. **(C)** Two-step photothermal ablation protocol combining intratumoral and transdermal irradiation. **(D)** Complete tumor elimination with no recurrence observed. Reprinted with permission from Muhanna et al. (2015). Copyright 2015 Ivyspring International.

The principle of multimodal nanoprobes combining multiple imaging modalities is to integrate probes of different imaging modalities onto a single nanoplatform (Kobayashi et al., 2007; Mallia et al., 2014; Ju et al., 2023). For instance, by integrating MRI probes and optical imaging probes onto one nanoplatform, multimodal imaging combining MRI and optical imaging can be achieved (Jing et al., 2014). Such multimodal imaging can improve the accuracy of defining tumor boundaries through the complementarity of different imaging information (Jing et al., 2014). Multimodal nanoprobes have made significant progress in preclinical studies of HNC(Muhanna et al., 2015). For example, Gang Zheng’s group has developed a multimodal nanoprobe (porphysomes) based on porphyrin-lipid, which can self-assemble into liposome-like nanoparticles (Figure 5) (Muhanna et al., 2015). Porphysomes demonstrate high stability in serum, with partial dissociation generates fluorescence, while the majority of porphysomes remain undegraded, making them highly effective for photoacoustic imaging. In the head and neck mice tumor model and rabbit tumor model, intravenous injection leads to clear visualization of primary tumors and metastatic lymph nodes via fluorescence and photoacoustic imaging 24 h later. Porphysomes enable local photothermal therapy (PTT) at tumor sites, effectively inhibiting tumor growth. With minimal barriers to clinical translation, these nanoparticles hold significant promise for providing precise and effective treatment options for patients with HNC. Its unique properties and multifunctionality position it as a promising candidate for advancing targeted cancer therapies.

## 3 The application of nanomaterials in surgical navigation for HNC

### 3.1 Fluorescent nanomaterials guiding surgical resection

**FIGURE 6:**
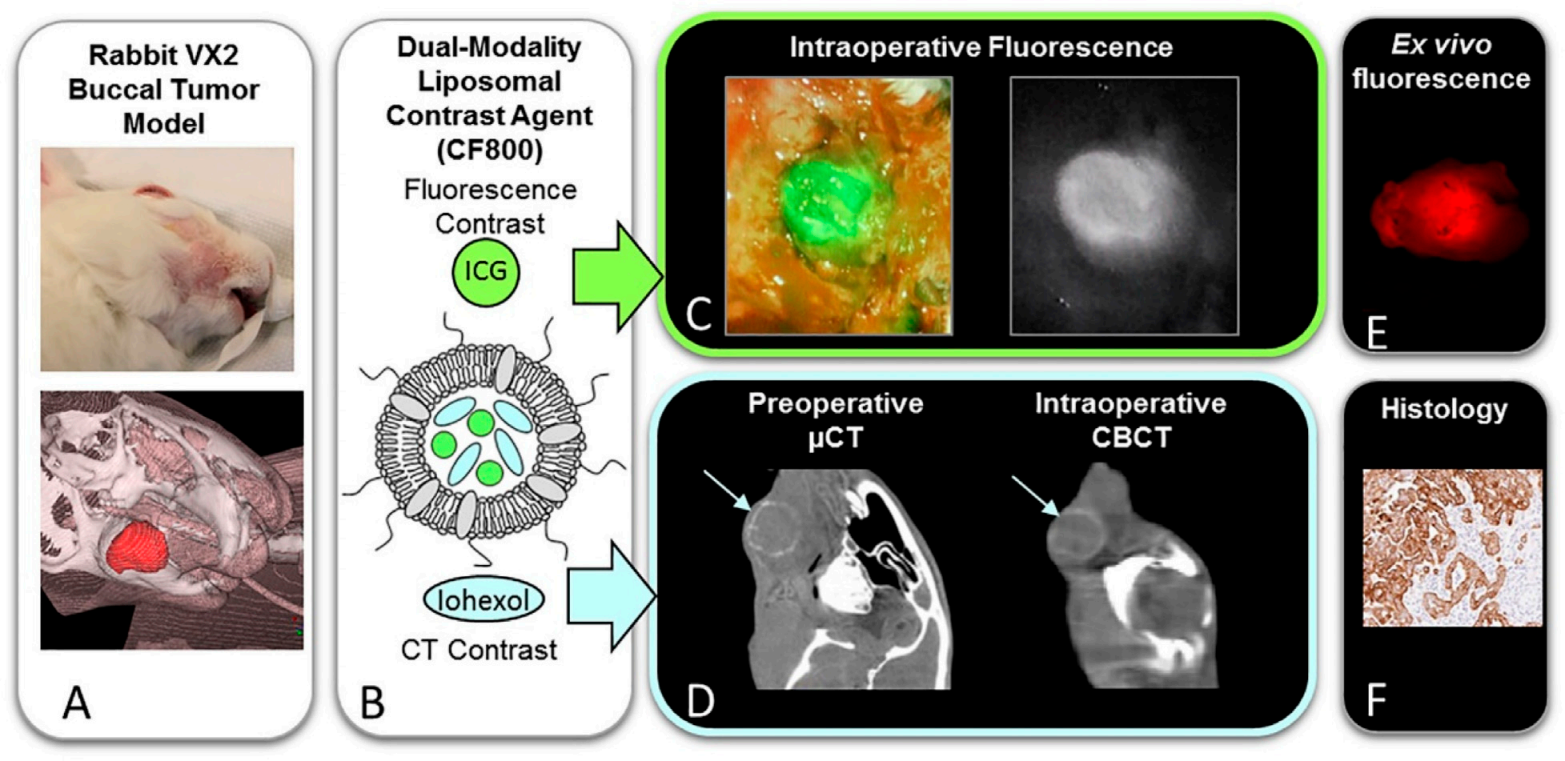
Schematic illustration of dual modality fluorescent/CT contrast liposomal agent (CF800) for surgical navigation. **(A)** Photographic and 3D reconstructed views of rabbit VX2 buccal carcinoma model. **(B)** Representation of CF800 dual-modality liposomal nanoparticles co-encapsulating indocyanine green (ICG) for near-infrared fluorescence imaging and iohexol-based computed tomography (CT) contrast. **(C)** Intraoperative near-infrared fluorescence imaging. **(D)** Preoperative micro-CT (μCT) and intraoperative cone-beam CT (CBCT). **(E)** Ex vivo tissue fluorescence quantification. **(F)** Histopathological validation.Reprinted with permission from (Muhanna et al., 2021) Copyright 2021 Elsevier.

Current imaging modalities are largely restricted to preoperative assessment, while intraoperative tumor margin determination still relies heavily on the surgeon’s tactile evaluation and frozen section analysis (Trotta et al., 2011). However, the efficacy of this method remains heavily dependent on the surgeon’s expertise and the tumor’s infiltrative growth pattern. To overcome these limitations, intraoperative imaging technologies, such as fluorescence-guided surgery (FGS) and cone-beam computed tomography (CBCT), are currently under investigation for real-time visualization of tumor margins (Curtin, 2012). FGS enhances detection accuracy of tumor margins through molecular-targeted fluorescence guidance, enabling real-time delineation of tumor boundaries. In contrast, CBCT enables intraoperative acquisition of real-time images, particularly offering advantages in surgeries involving complex bony structures such as the skull base, mandible, and maxilla (Trotta et al., 2011; Curtin, 2012). There exists a variety of fluorescent nanoparticles applicable to surgical navigation, such as persistent luminescent nanoparticles and the NIR fluorescence sensor (Craig et al., 2016; Yin et al., 2024; Zhong et al., 2024). The mechanism of specific accumulation of fluorescent nanoparticles in HNC tissues mainly depends on the specific binding between targeting molecules and surface markers of cancer cells. Under the excitation by near-infrared light, fluorescent nanoparticles emit fluorescent signals, which can be observed using fluorescent microscopes, endoscopes, and other devices. By real-time observation the distribution of fluorescent signals, the surgical resection process can be guided to ensure the complete excision of tumor tissues. Jonathan C. iris constructed a novel multi-modal liposomal contrast agent (CF800) to enable three-dimensional localization and soft-tissue visualization (Figure 6) (Muhanna et al., 2021). Significant enhancement in CBCT images was observed starting 24 h after CF800 administration, peaking at 96 h, and remaining notable at 120 h. The long half-life of CF800 supports its application for multiple intraoperative imaging sessions. Fluorescence-guided surgery enables real-time visualization of tumor boundaries, thereby enhancing surgical precision. Additionally, CF800 demonstrates a high signal-to-noise ratio in *ex vivo* imaging, which aids in the accurate assessment of surgical margins. These features collectively highlight CF800s potential to improve both intraoperative decision-making and postoperative evaluation in oncological surgeries.

### 3.2 Magnetic reso-nance nanoprobe guiding surgical resection

Despite continuous advancements in oncological surgery, microscopic residual disease (MRD) remains a significant factor contributing to cancer recurrence and metastasis (Ferrero et al., 2012; Sano et al., 2012). This issue is particularly common in aggressive cancers such as HNC (Sano et al., 2012). Current clinical methods, including palpation and imaging, lack the sensitivity to detect MRD effectively (Meier et al., 2005). Pathological analysis of surgical margins, while commonly used, is often slow and inaccurate. To eliminate potential MRD, surgeons frequently resort to removing large volumes of normal tissue (Loree and Strong, 1990; de Carvalho et al., 2012). This approach not only fails to guarantee complete tumor removal but also increases patient morbidity, reduces quality of life, and complicates postoperative treatments such as radiotherapy and chemotherapy. Moreover, MRD often develops resistance to these therapies, further diminishing patient survival rates.

Emerging diagnostic technologies have yet to achieve single-cell sensitivity and real-time detection of MRD in solid tissues (Mito et al., 2012). Optical methods, for instance, are limited to detecting larger surface tumors, while photoacoustic techniques lack sensitivity, speed, and specificity in solid tissues. Fluorescence methods lack the necessary sensitivity, and multispectral optoacoustic tomography cannot detect small clusters of cancer cells in real time within solid tissues (Razansky et al., 2011). Even when MRD is identified through frozen section pathology, standard surgical procedures often struggle to remove it without damaging critical organs (Chen and Karlan, 2000). These limitations highlight the critical need for novel nanomaterials enabling precise intraoperative detection and management of MRD.

**FIGURE 7:**
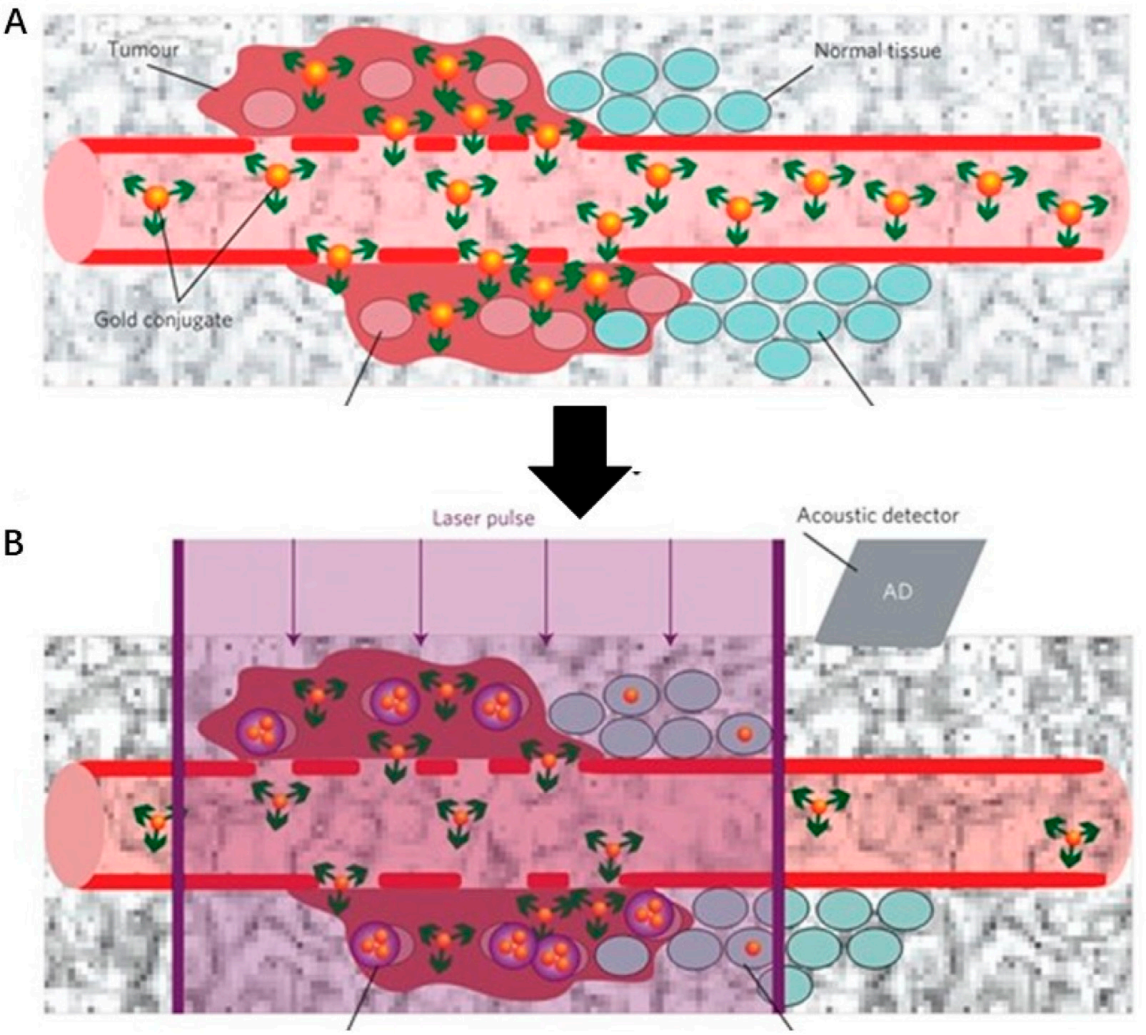
Schematic illustration of a plasmonic nanobubble (PNB) surgical technology for the intraoperative management **(A)** Systemic delivery of gold conjugates to the tumour *via* their leaky vasculature **(B)** The acoustic signal of a PNB (illustrative red time response) reports even a single cancer cell in solid tissue, but not normal cells (illustrative green time response). Reprinted with permission from Lukianova-Hleb et al. (2016). Copyright 2016 Springer Nature.

Ekaterina Y. Lukianova-Hleb and colleagues have developed a plasmonic nanobubble (PNB) surgical technology for the intraoperative management of both resectable and unresectable tumors (Figure 7) (Lukianova-Hleb et al., 2016). This approach involves the local detection and resection of resectable MRD, as well as the mechanical destruction of unresectable MRD using cancer cell-specific PNBs. By combining systemic gold nanoparticle targeting, localized PNB generation with low-energy laser pulses, and acoustic detection, this technology offers a promising solution for real-time, *in vivo* detection and elimination of MRD during surgery. The high sensitivity and specificity of PNBs, combined with their compatibility with standard surgical protocols, could significantly improve surgical outcomes while minimizing morbidity.

### 3.3 Nanotechnology-enhanced surgical visualization

Currently, researchers are developing advanced imaging technologies to achieve preoperative visualization of HNC(Heidari et al., 2020; Orosz et al., 2020). Among these, MRI is the preferred modality for evaluating primary tumors in the head and neck region due to its high spatial resolution for soft tissue structures (Freihat et al., 2020; Wu et al., 2021). However, the large size of MRI equipment and lengthy acquisition times limit its applicability as an intraoperative imaging guidance instrument (Radtke et al., 2016; Yip and Aerts, 2016; Arnold et al., 2022). In contrast, optical imaging methods offer several advantages, including high sensitivity, relatively low cost, and widespread availability (Gröhl et al., 2021; Park et al., 2023; Zhu et al., 2024). By taking advantage of the strengths of different imaging modalities, nanotechnology enables surgeons to more precisely delineate tumor margins both before and during surgery, thereby improving the completeness of tumor resection, particularly for HNC.

**FIGURE 8:**
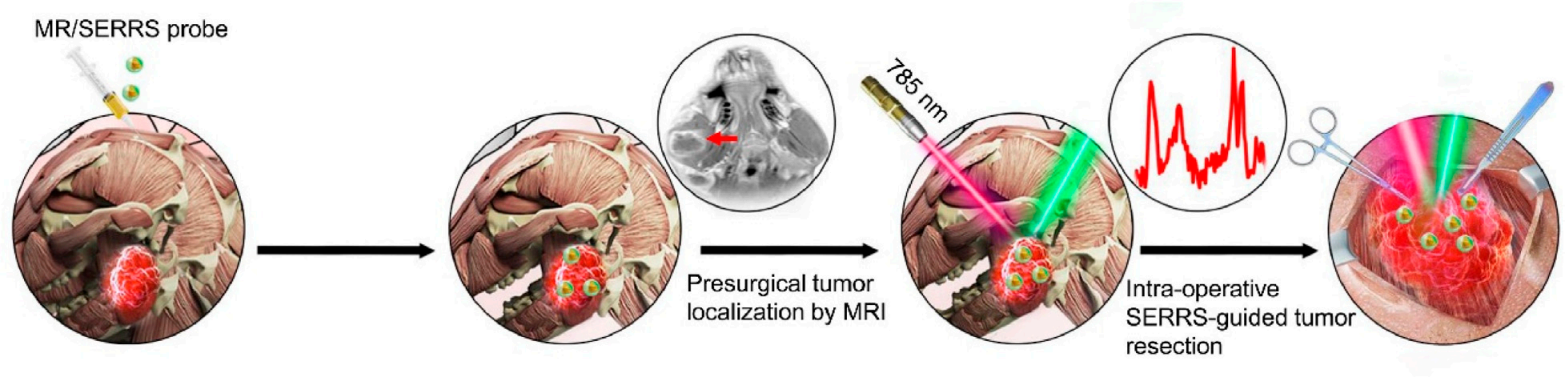
Schematic illustration of the multifunctional MRI-SERRS nanoprobe (AuS-Cy7-Gd) for the intraoperative guided surgery. Reprinted with permission from Sun et al. (2019). Copyright 2019 Elsevier.

Surface-enhanced Raman scattering (SERS) technology, which uses roughened metallic surfaces as enhancing substrates, can boost Raman signals by a factor of 10^6^ to 10^10^ compared to traditional Raman techniques (Hu et al., 2017b; Lin et al., 2023; Zhu et al., 2024). In addition to its exceptional signal enhancement, SERS offers multiplexing capabilities, high sensitivity, and enhanced photostability, making it a powerful tool for detecting molecular biomarkers and diagnosing tumors (Qiao et al., 2018; Kaur et al., 2022; Zhang et al., 2022). By employing molecular reporters with absorption energies similar to the incident laser, surface-enhanced resonance Raman spectroscopy (SERRS) further enhances signals by an additional 10^2^ to 10^3^ times (Gao and Weaver, 1987; Zong et al., 2018; Plou et al., 2022). As a result, SERRS not only inherits the advantages of SERS but also achieves significantly higher sensitivity, demonstrating immense potential for guiding tumor resection (Plou et al., 2022). The extremely high sensitivity of SERRS allows for real-time delineation of infiltrative tumor margins, while MRI excels in preoperative tumor localization and preliminary malignancy assessment. The combination of SERRS and MRI thus holds unprecedented potential for guiding tumor resection, such as in gliomas, enabling more precise and complete removal of tumor tissue. Xiaofeng Tao’s group developed a multifunctional MRI-SERRS nanoprobe (AuS-Cy7-Gd) (Sun et al., 2019), which combines the strengths of SERRS and MRI to improve the surgical management of HNC (Figure 8). The AuS-Cy7-Gd specifically accumulates in tumor tissues through the enhanced permeability and retention (EPR) effect, contributing to both preoperative MRI and intraoperative SERRS-guided surgery. SERRS-guided surgery enables precise identification of tumor margins and metastatic lymph nodes, reducing residual tumor tissue and enhancing the completeness of surgical resection, thereby prolonging patient survival.

## 4 Application of nanomaterials in the postoperative adjuvant treatment of HNC

Surgical resection is widely regarded as the primary treatment option for HNC in clinical practice (Hamoir et al., 2018; Cramer et al., 2019; Mody et al., 2021). However, approximately 40%–60% of patients with advanced HNC experience local recurrence postoperatively, primarily due to the difficulty in completely eliminating residual tumor cells at the resection margins (Mahvi et al., 2018). Additionally, postoperative patients may develop localized immunosuppression, further aggravating the risk of recurrence (Mahvi et al., 2018). To reduce postoperative tumor recurrence, conventional chemotherapy and radiotherapy are frequently applied (Qiao et al., 2018; Bu et al., 2019; Petit et al., 2021); however, these approaches are limited by systemic toxicity, drug resistance, and immune system damage (Petit et al., 2021). In recent years, immune checkpoint blockade (ICB) antibody-based immunotherapy has attracted significant attention due to its promising long-term outcomes in preventing tumor recurrence (Ruan et al., 2019; Dolladille et al., 2020). ICB antibodies, such as programmed cell death protein 1 (PD-1) and its ligand programmed death-ligand 1 (PD-L1), have been proposed to alleviate the immunosuppressive tumor microenvironment (TME) (Kurtulus et al., 2019; Schnell et al., 2020). Despite their clinical efficacy, ICB therapies face several challenges, including poor tumor immunogenicity and immune-related systemic side effects (Chen et al., 2019; Kennedy and Salama, 2020; Zhou et al., 2021). Since the effectiveness of ICB therapy largely depends on sufficient tumor-infiltrating T Cells, converting “cold” tumors into immunogenic “hot” tumors represents a promising strategy to enhance immunotherapy outcomes (Chen et al., 2019; Chao et al., 2020). Furthermore, improving methods to leverage the immune system for localized delivery of immunotherapeutic agents may be crucial to stimulate robust antitumor immune responses and prevent local tumor recurrence. Photothermal therapy (PTT) is a non-invasive treatment modality that uses NIR light to irradiate photothermal agents, generating high thermal energy to eliminate tumor cells (Liu R. et al., 2023; Overchuk et al., 2023). Recent studies have demonstrated that PTT ablation can induce immunogenic cell death (ICD) in tumor cells, leading to the release of a wide range of tumor-associated antigens, damage-associated molecular patterns (DAMPs), and pro-inflammatory cytokines (Duan et al., 2018; Ma et al., 2019; Meier et al., 2024). Recent evidence suggests that a newly identified form of ICD, plays a significant role in enhancing antitumor immunity (Wang et al., 2017). Pyroptosis is characterized by cell swelling and the release of cellular contents, which further enhance immune responses (Wang et al., 2017; Xiao et al., 2021; Du et al., 2024). These findings highlight the potential of combining PTT with pyroptosis-inducing strategies to improve therapeutic outcomes by fostering a robust antitumor immune environment.

**FIGURE 9:**
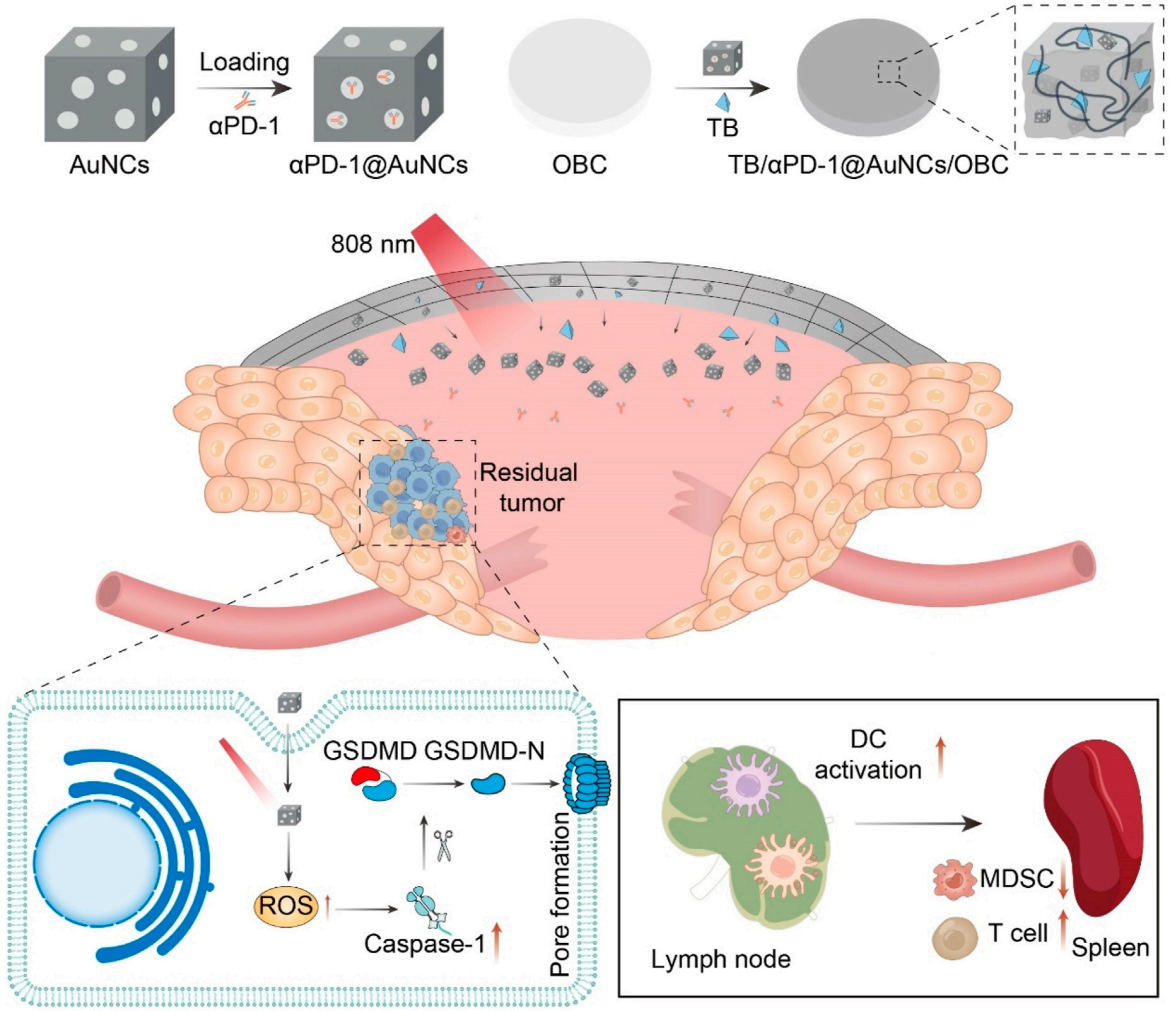
Schematic illustration of the versatile OBC-based membrane for achieving a therapeutic effect in antitumor immunotherapy towards HNC postoperative treatment.

Our group rationally constructed a versatile implant (TB/αPD-1@AuNCs/OBC) for HNC tumor postoperative treatment (Figure 9) (Zhou et al., 2022). The TB/αPD-1@AuNCs/OBC under NIR irradiation allowed the combination of therapies with remarkable postoperative antitumor immunity to reduce locoregional recurrence and improve survival. We rationally constructed a versatile implant (TB/αPD-1@AuNCs/OBC) for HNC tumor postoperative treatment. The TB/αPD-1@AuNCs/OBC under NIR irradiation allowed the combination of therapies with remarkable postoperative antitumor immunity to reduce locoregional recurrence and improve survival.

## 5 Advantages of nanomaterials application in the perioperative period of HNC

### 5.1 Accurate targeted therapy for enhanced treatment efficacy

Nanomaterials achieves the precise identification of HNC and drug delivery by modifying nanocarriers with targeting molecules (Souza et al., 2019; Zhang et al., 2023; Tang et al., 2024). This precision-targeted therapy can increase the concentration of therapeutic drugs in tumor tissues and reduce damage to normal tissues, thus enhancing the treatment efficacy (Xu et al., 2015; Lee et al., 2018; Souza et al., 2019). In the treatment of HNC, precision-targeted treatment allows drugs to act more concentratedly on tumor cells, strengthening the killing effect on tumor cells while avoiding negative effects on normal tissues (Kitamura et al., 2020). For example, some studies have shown that targeted nano-drug delivery systems can significantly enhance the accumulation of chemotherapy drugs in tumor tissues and improve the uptake efficiency of tumor cells for drugs, thereby increasing the treatment effectiveness rate (Bhirde et al., 2009; Li et al., 2016; Hu et al., 2018; Song et al., 2020). Nanomaterial-mediated multimodal treatment, through the synergy of different treatment mechanisms, can more effectively kill tumor cells, inhibit tumor growth, recurrence, and metastasis, further improving the treatment outcome of HNC(Li et al., 2019; Zhou et al., 2022; Hu et al., 2024). Taking the combination of chemotherapy and photothermal therapy as an example (Nam et al., 2018; Wang et al., 2019; Li et al., 2023), the high temperature generated by photothermal treatment can disrupt the integrity of the tumor cell membrane and increase its permeability, enabling chemotherapy drugs to enter the interior of tumor cells more easily and enhancing the cytotoxic effect of chemotherapy drugs. Meanwhile, chemotherapy drugs can kill tumor cells that are insensitive to photothermal treatment. The two treatments cooperate with each other to form a synergistic effect and improve the killing efficiency of tumor cells. This synergy has been verified in experimental studies on HNC treatment, demonstrating a more superior treatment effect than single treatment modalities (Wang et al., 2019).

### 5.2 Reduce the incidence of complications

Nanomedicine carriers achieve the slow and continuous release of drugs in tumor tissues by controlling the drug release rate and site, which lowers the peak blood drug concentration and mitigates the toxic side effects of drugs on normal tissues throughout the body, thereby improving patients’ tolerance to treatment (Qiu et al., 2018; Moradi Kashkooli et al., 2020; Liu et al., 2024). For example, Poly (lactic-co-glycolic acid) (PLGA) nanoparticles have been extensively used for the delivery of anticancer drugs. However, their application is often limited by challenges such as rapid clearance, burst release, and uncontrolled drug diffusion into surrounding tissues. Chitosan/β-glycerophosphate (CS/GP) thermosensitive hydrogels, known for their biocompatibility and biodegradability, have emerged as a promising platform for drug delivery and tissue engineering. By encapsulating drug-loaded PLGA nanoparticles within CS/GP hydrogels, the solubility and bioavailability of the therapeutic agents can be significantly enhanced. This hybrid system enables sustained and controlled drug release, improves local drug delivery and retention, and consequently enhances the therapeutic efficacy in HNC.

This approach represents a significant advancement in overcoming the limitations of conventional nanoparticle-based drug delivery systems to enhance the treatment effect while reducing the adverse impacts on patients’ bodies.

### 5.3 Improve the patients’ quality of life

Nanotechnology contributes to more precise tumor resection and better preservation of oral organ functions by providing real-time and accurate information on tumor boundaries and surgical guidance. It reduces damage to surrounding normal tissues such as nerves and blood vessels (Ghosh et al., 2014; Jin et al., 2016; Zhu et al., 2022). For example, in HNC surgery, the use of fluorescent nanomaterials to mark the tumor boundaries enables surgeons to clearly visualize the extent of the tumor during the operation, preventing accidental resection of normal surrounding tissues (Lukianova-Hleb et al., 2016). This precise surgical operation not only increases the success rate of the surgery but also may decrease the occurrence of postoperative complications. Meanwhile, better preservation of oral organ functions is of great significance for patients’ quality of life. Patients can maintain better masticatory, swallowing, and speech functions after the operation, enhancing their self-care ability and quality of life.

## 6 Nanomaterials application challenges in HNC peri-operative period

### 6.1 Safety of nanomaterials

The metabolic pathways and distribution of nanomaterials within the human body are rather complex following administration (Zhao and Castranova, 2011; Navya et al., 2019; Hussain et al., 2020). Some nanomaterials may accumulate in organs such as the liver and spleen, and long-term accumulation might potentially affect the functions of these organs (Hussain et al., 2020). For example, certain metal nanoparticles could trigger an inflammatory response in the liver and impact its normal metabolic function (Noga et al., 2023). In addition, nanomaterials may also enter other tissues and organs through the bloodstream, causing damage to them (Attarilar et al., 2020; Sengul and Asmatulu, 2020). Research has shown that the physicochemical properties of nanomaterials, such as size, shape, and surface charge, can influence their metabolism and distribution in the body (Haripriyaa and Suthindhiran, 2023). Nanomaterials with a smaller size may more easily penetrate biological membranes and enter the interior of cells, thus affecting the physiological functions of cells. Evaluating the safety of nanomaterials demands a deep understanding of their toxicity mechanism (Li et al., 2022). The toxicity of nanomaterials may stem from their interactions with biomolecules, leading to cytotoxicity, inflammatory responses, oxidative stress, etc (Li et al., 2022). For instance, the charge on the surface of nanomaterials may interact with the charge on the cell membrane surface, resulting in an increase in the permeability of the cell membrane, allowing the leakage of intracellular substances and subsequently affecting the normal physiological functions of cells. Moreover, nanomaterials may also react with biomolecules such as intracellular proteins and nucleic acids, interfering with their normal structures and functions. Some nanomaterials may induce cells to generate oxidative stress responses, producing a large amount of reactive oxygen species (ROS) (Li et al., 2014). These ROS will cause damage to the cell’s DNA, proteins, and lipids, leading to cell apoptosis or necrosis (Li et al., 2014).

### 6.2 Standardization and normalization of nanomaterials

Currently, there are various methods for preparing nanomaterials, such as physical, chemical, and biological methods (Forsberg, 2011; Abram et al., 2025). Different preparation methods result in variations in the properties of nanomaterials (Forsberg, 2011; Abram et al., 2025). For example, nanomaterials prepared by physical methods may possess good crystallinity and high purity, but the particle size distribution might be relatively wide. Nanomaterials prepared by chemical methods can precisely control the particle size and shape, yet some impurities may be introduced. These differences affect the application efficacy of nanomaterials in the treatment of HNC. Therefore, it is necessary to compare and evaluate different preparation methods to select the most suitable one for the treatment of HNC.

## 7 Conclusion and outlook

Future multifunctional nanomaterials are expected to combine diagnosis, treatment, and monitoring into a single platform. For instance, nanoplatforms could integrate chemotherapeutic drugs, tumor-targeting ligands, and fluorescence imaging agents to achieve precise diagnosis, personalized therapy, and real-time treatment monitoring for HNC. Such designs align with recent advances in soft monomicelle-directed synthesis, where amphiphilic block copolymers enable precise control over nanoparticle architectures for tailored drug delivery and imaging functionalities. (Wang J. et al., 2024). These platforms could further incorporate redox-responsive linkages, as demonstrated by disulfide-bridged prodrug systems (MTX@LND NPs), which release drugs selectively in tumor microenvironments *via* glutathione (GSH)-triggered disassembly (Hua et al., 2024). By combining these strategies, multifunctional nanoplatforms can synergize chemotherapy, targeted delivery, and imaging, reducing treatment costs and improving patient outcomes.

Moreover, with the continuous development of gene detection technology, it is anticipated that genotyping will be combined with nanomaterials in the future to provide more personalized treatment protocols for patients with HNC. Genotyping-guided nanomedicine could leverage protein-based nanoparticles, which offer inherent biocompatibility and customizable surfaces for tumor-specific targeting (Liu H. et al., 2023). For example, albumin nanoparticles modified with molecular markers (such as EGFR, CD44) could deliver drug combinations optimized for individual genetic profiles, minimizing off-target effects. This approach mirrors the precision of redox-responsive nanosystems, where stimuli-triggered release enhances therapeutic specificity (Hua et al., 2024). By personalizing nanomedicines, the effectiveness and safety of treatment can be enhanced, and the occurrence of adverse reactions can be reduced.

The profound integration of nanomaterials with medical imaging and bioengineering will bring more innovative solutions to the perioperative treatment of HNC. In the field of imaging, nanomaterials can be used to develop novel nano-imaging probes such as protein-based MRI contrast agents (Hua et al., 2024), enhancing the diagnostic accuracy and visualization of tumors. In the aspect of engineering, nanomaterials can be combined with tissue engineering to develop nanomaterials and scaffolds that are more suitable for the repair and regeneration of HNC issues. The perioperative treatment of HNC involves multiple disciplinary fields, including oral and maxillofacial surgery, medical oncology, radiology, nanomaterials experts, bioengineers, *etc.* Multidisciplinary team collaboration is essential to optimize nanomaterials applications in HNC perioperative care. Through the close collaboration of interdisciplinary teams, the advantages of each field can be fully used to jointly formulate more scientific and reasonable treatment plans. For instance, oral and maxillofacial surgeons can put forward requirements for nanomaterials based on surgical needs. Nanomaterials experts can design responsive nanomaterials using techniques like photo-reduction synthesis (Hua et al., 2024) or redox-sensitive assembly (Liu H. et al., 2023) according to these requirements. Medical oncologists can propose the design ideas of nano-drugs in combination with the characteristics of chemotherapy drugs. Radiologists can evaluate the treatment effect by using imaging techniques. This multidisciplinary team cooperation model will help accelerate the translation of nanomaterials from basic research to clinical practice and improve the treatment effect of patients with HNC.

In summary, nanomaterials have demonstrated remarkable application potential at every stage of HNC perioperative management (Table 1). In the preoperative diagnosis, the application of nanoprobes in defining tumor boundaries has enhanced the accuracy of diagnosis and the ability to detect tumors at an early stage, providing more precise information for surgical treatment. During the surgical navigation, the use of fluorescent nanomaterials and nanotechnology-assisted surgical instruments has improved the precision and visualization of the surgery, reducing the damage to surrounding normal tissues. In the postoperative adjuvant treatment, the targeted nano drug delivery systems and the combined treatment mediated by nanomaterials have increased the treatment efficacy and decreased the incidence of complications. With the continuous development and innovation of nanomaterials, the future treatment of HNC will show a trend towards more personalized, precise, and efficient approaches. The research and development of new nanomaterials and technologies will offer more options and possibilities for the diagnosis and treatment of HNC. The optimization of the multidisciplinary combined treatment model will integrate the advantageous resources of various fields to provide more comprehensive and integrated treatment services for patients. It is believed that in the near future, nanomaterials will play a more crucial role in the perioperative treatment of HNC, bringing better treatment outcomes and quality of life to patients.

**TABLE 1:** Representative nanomaterials summarized in this review for different stages of perioperative HNC treatment.

Stage	Materials	*In vitro*/vivo model	Therapeutics	References
Preoperation	QD800-EGFR	BcaCD885 cells BcaCD885 mice model	Visualization Imaging	Yang (2011)
	PCA-LDA	OSCC patients’ blood serum samples	Preoperative Pathological Assessment	Xue et al. (2018)
	RB-GNR	CAL-27 cells	Preoperative Diagnosis	Wang et al. (2013)
	Porphysomes	HNC hamster models/VX2 HNC rabbit model	Defining the Tumor Boundary	Muhanna et al. (2015)
Intraoperation	CF800	VX2 HNC rabbit model	Guiding Surgical Resection	Muhanna et al. (2021)
	PNB	HN31 HNSCC mice model	Guiding Surgical Resection	Lukianova-Hleb et al. (2016)
	AuS-Cy7-Gd	VX2 HNC rabbit model	Surgical Visualization	Sun et al. (2019)
Postoperation	TB/αPD-1@AuNCs/OBC	SCC7 HNSCC mice model	Postoperative Reduce Recurrence	Zhou et al. (2022)
